# Role of spike compensatory mutations in the interspecies transmission of SARS-CoV-2

**DOI:** 10.1016/j.onehlt.2022.100429

**Published:** 2022-08-29

**Authors:** Roger Frutos, Nouara Yahi, Laurent Gavotte, Jacques Fantini, Christian A. Devaux

**Affiliations:** a Cirad, UMR 17, Intertryp, Montpellier, France; b INSERM UMR_S 1072, Aix-Marseille Université, 13015 Marseille, France; c Université de Montpellier, UMR Espace-Dev, Montpellier, France; d Aix-Marseille Université, IRD, APHM, MEPHI, IHU–Méditerranée Infection, Marseille, France; e CNRS, CNRS-SNC5039, Marseille, France

**Keywords:** COVID-19 vaccine, SARS-CoV-2 variants, Mink, hamster, Coronavirus, ACE2

## Abstract

SARS-CoV-2, the virus responsible for COVID-19 in humans, can efficiently infect a large number of animal species. Like any virus, and particularly RNA viruses, SARS-CoV-2 undergoes mutations during its life cycle some of which bring a selective advantage, leading to the selection of a given lineage. Minks are very susceptible to SARS-CoV-2 and owing to their presence in mass rearing, they make a good model for studying the relative importance of mutations in viral adaptation to host species. Variants, such as the mink-selected SARS-CoV-2 Y453F and D614G or H69del/V70del, Y453F, I692V and M1229I were identified in humans after spreading through densely caged minks. However, not all mink-specific mutations are conserved when the virus infects human populations back. Many questions remain regarding the interspecies evolution of SARS-CoV-2 and the dynamics of transmission leading to the emergence of new variant strains. We compared the human and mink ACE2 receptor structures and their interactions with SARS-CVoV-2 variants. In minks, ACE2 presents a Y34 amino acid instead of the H34 amino acid found in the human ACE2. H34 is essential for the interaction with the Y453 residue of the SARS-CoV-2 Spike protein. The Y453F mink mutation abolishes this conflict. A series of 18 mutations not involved in the direct ACE2 interaction was observed in addition to the Y453F and D614G in 16 different SARS-CoV-2 strains following bidirectional infections between humans and minks. These mutations were not random and were distributed into five different functional groups having an effect on the kinetics of ACE2-RD interaction. The interspecies transmission of SARS-CoV-2 from humans to minks and back to humans, generated specific mutations in each species which improved the affinity for the ACE2 receptor either by direct mutation of the core 453 residue or by associated compensatory mutations.

## 1 Introduction

In December 2019 a Betacoronavirus/Sarbecovirus, later on named SARS-CoV-2, was found associated with a severe respiratory disease affecting people hospitalized in China [[1], [2], [3]]. This disease currently known as COVID-19 (Coronavirus Disease-2019), can sometimes lead to severe forms and to death but in the large majority of cases the infection remains asymptomatic or induces only moderate Influenza-like symptoms that can be treated with appropriate medical care [4,5]. The current estimated mean case fatality rate of COVID-19 is ranging between 1% and 2% [6]. However, this rate might be overestimated since asymptomatic cases are not considered.

A virus identical to the first SARS-CoV-2 characterized in human was never found in wild animals despite numerous studies and sampling programs. Only related viruses belonging to differing populations were found in various bat species and in pangolin, the species that have been the subject of the deepest investigations. However, SARS-CoV-2 was shown to be able to infect many animal species present in the direct vicinity of humans [7] such as either domestic animals like cats [[8], [9], [10]], animals in zoos [11,12] and in rearing like minks or hamsters [[13], [14], [15]] or in contact with humans like white-tailed deer [16]. Minks, whether it is the American mink (*Neovison vison*) or the European mink (*Mustela lutreola*), make a good model for studying the interspecies transmission and evolution of SARS-CoV-2 [17]. Human-mink bidirectional cross-infections were shown to easily occur with an initial contamination event being from humans to minks [[17], [18], [19]]. A SARS-CoV-2 outbreak in mink (*N. vison*) farms in Denmark was characterized by a rapid spread of the virus [13]. SARS-CoV-2 infections were reported in hundreds of farms in Europe and North America. About 170 mutations were identified in the genome of SARS-CoV-2 spreading in minks [20]. Although many of the mink SARS-CoV-2 genomes were similar to those of humans, specific mutations were detected in mink SARS-CoV-2 which indicates an adaptation during viral transmission between minks [17]. These mutations in the SARS-CoV-2 genome could be selected to confer the virus a capacity to escape a specific host immune response or to overcome intracellular mechanisms underlying the inhibition of the viral replication cycle. Variants deriving from minks were apparently less virulent in humans [21]. The combination of the Y453F mutation in minks with H691, V701, I692V, and M1229I reduced the capacity of entry into some cell lines [22].

An abundant literature on ACE2 species polymorphism highlighted that dozens of animal species could be susceptible to SARS-CoV-2 infection [[23], [24], [25], [26], [27], [28], [29], [30], [31]]. In silico analysis indicated that mink ACE2 proteins from both *Mustela erminae* and *Mustela putorius furo*, could serve as receptors for SARS-CoV-2 [25,26,31]. The investigation of SARS-CoV-2 outbreaks in Netherlands mink (*M. lutreola*) breeding farms revealed that the virus was introduced into farms by humans followed by transmission among minks [[17], [18], [19]]. Similarly, the investigation of SARS-CoV-2 outbreak in mink (*N. vison*) breeding farms in Denmark showed a rapid spread of the virus [13]. Some 170 mutations were identified in the genome of SARS-CoV-2 spreading in minks [20]. Building on the availability 3-D structures of both the viral spike (S) protein and ACE2 [22,[32], [33], [34], [35]], we addressed in this work the role of adaptation to ACE2 polymorphism in the selection of host-specific SARS-CoV-2 variants and the dynamics of interspecies viral transmission.

## 2 Material and methods

ACE2 Protein Sequence. The protein sequences from 23 different species were collected from Genbank. These sequences are the following: *Homo sapiens* (BAB40370), *Macaca mulatta* (NP_001129168), *Felis catus* (AAX59005), *Panthera tigris* (XP_007090142), *N. vison* (QPL12211), *Mustela nigripes* (QNC68914), *M. putorius furo* (NP_001297119), *M. lutreola* (QNC68911), *Mustela erminae* (XP_032187679), *Paguma larvata* (AAX63775), *Sus scrofa* domestic (ASK12083), *Sus scrofa* (NP_001116542), *Rhinolophus affinis* (MT394227), *Rhinolophus affinis* (MT394223), *Rhinolophus macrotis* (ADN93471), *Rhinolophus sinicus* (AGZ48803), *Rhinolophus pearsonii* (ABU54053), *Manis javanica* (XP_017505752), *Rattus rattus* (XP_032746145), *Mus musculus* (NP_081562), *Gallus gallus* (QEQ50331), *Pelodiscus sinensis* (XP_006122891), *Xenopus tropicalis* (XP_002938293), and *Ophiophagus hannah* (ETE61880).

Comparison of ACE2 sequences. ACE2 sequences were aligned and compared using the Clustal Omega multiple sequence alignment (EMBL-EBI bioinformatic tool; Copyright © EMBL 2020) (https://www.ebi.ac.uk/Tools/msa/ clustalo/).

3-D analysis of ACE2 and ACE2-viral spike complexes. All-atom in-silico analyses were performed using Hyperchem (http://www.hypercubeusa.com), Deep View/Swiss-Pdb viewer (https://spdbv.vital-it.ch) and Molegro Molecular viewer (http://molexus.io/molegro-molecular-viewer) as previously described [36]. The initial coordinates were obtained from pdb 6m0j [33]. The structure was then submitted to several rounds of energy minimization with the Polak-Ribière algorithm according to our standardized protocol [36]. This source file model was used to introduce the indicated mutations in the Receptor Binding Domain (RBD) with the MUTATE tool of Swiss-PdbViewer [37]. ACE2 polymorphisms were also modulated from this source file with a similar approach. Docked complexes were subsequently submitted to iterative cycles of energy minimization under the CHARMM molecular force field with the convergence condition of root mean square (RMS) gradient less than 0.01 kcal. Å^−1^.mol^−1^ [38]. Interaction energies were calculated from stable complexes using the Ligand Energy Inspector function of Molegro. The electrostatic potential measured and illustrated by Molegro Molecular viewer is the sum of the Coulomb potentials for each atom of the considered molecule, with a distance-dependent dielectric constant. Colour intensities of the electrostatic surface potential were quantified with the ImageJ software [36]. Our in silico approaches have been previously validated with structural studies of several SARS-CoV-2 variants including Omicron BA.1. Indeed, the heavily mutated RBD structure predicted by our in silico analysis shortly after the emergence of this variant was remarkably similar to posterior cryo-electron microscopy data (pdb 7t9l) [39,40].

SARS-CoV-2 spike gene and protein sequence analysis. SARS-CoV-2 spike sequences from humans, European minks and American minks were collected from Genbank and GISAID (Table 1) [17,41]. Multiple alignments were conducted with MUSCLE using the SeaView package [42]. The nucleic acid phylogenetic tree was built the using the maximum likelihood method under the HKY model with 500 bootstrap repeats. The protein tree was built with the maximum likelihood method using the Q.plant +G + F model with 500 bootstrap repeats. Tress were rooted using the SARS-CoV-2 original Wuhan HU1 strain spike gene or protein (NC_045512.2) as outgroup.

**Table 1:** Sequences carrying compensatory mutations analyzed in this work.

Sequence name	Accession number	Mutations	Collection date	Country	Host
EPI_ISL_615543_Hsap_Nvis_DK^a^	EPI_ISL_615543	Y453F + D614G	07/09/2020	Denmark (DK)	Human + Mink
EPI_ISL_615652_Hsap-Nvis_DK	EPI_ISL_615652	Y453F + D614G + I692V + M1229I	14/09/2020	Denmark (DK)	Human + Mink
EPI_ISL_615996_Hsap_DK	EPI_ISL_615996	Y453F + D614G + E1031D	24/08/2020	Denmark (DK)	Human
EPI_ISL_616945_Hsap_DK	EPI_ISL_616945	Y453F + D614G + K814N	26/10/2020	Denmark (DK)	Human
EPI_ISL_616971_Hsap_Nvis_DK	EPI_ISL_616971	Y453F + D614G + S1147L	26/10/2020	Denmark (DK)	Human + Mink
EPI_ISL_617056_Hsap_DK	EPI_ISL_617056	Y453F + D614G + P681R	26/10/2020	Denmark (DK)	Human
EPI_ISL_618946_Hsap_Nvis_DK	EPI_ISL_618946	Y453F + D614G + N751Y	12/10/2020	Denmark (DK)	Human + Mink
EPI_ISL_619487_Hsap_DK	EPI_ISL_619487	Y453F + D614G + S1252P	12/10/2020	Denmark (DK)	Human
EPI_ISL_619643_Hsap_DK	EPI_ISL_619643	Y453F + D614G + T827S	12/10/2020	Denmark (DK)	Human
EPI_ISL_620233_Hsap_DK	EPI_ISL_620233	V308L + Y453F + D614G	05/10/2020	Denmark (DK)	Human
EPI_ISL_641413_Nvis_DK	EPI_ISL_641413	L5F + Y453F + D614G + N751Y + C1250F	22/10/2020	Denmark (DK)	Mink
EPI_ISL_618474_Hsap_DK	EPI_ISL_618474	Y453F + V534F + D614G	12/10/2020	Denmark (DK)	Human
EPI_ISL_621008_Hsap_DK	EPI_ISL_621008	Y453F + D614G + M731I	28/09/2020	Denmark (DK)	Human
EPI_ISL_625708_Hsap_DK	EPI_ISL_625708	Y453F + Q580H + D614G	26/10/2020	Denmark (DK)	Human
EPI_ISL_625720_Hsap_DK	EPI_ISL_625720	Y453F + D614G + A626S + N751Y	26/10/2020	Denmark (DK)	Human
EPI_ISL_625856_Hsap_DK	EPI_ISL_625856	Y453F + E583D + D614G	26/10/2020	Denmark (DK)	Human
EPI_ISL_641474_Hsap_Nvis_DK	EPI_ISL_641474	Y453F + D614G + D1139Y	09/09/2020	Denmark (DK)	Human + Mink

a) Reference sequence to which the sequences with compensatory mutations are compared.

## 3 Results

**Fig. 1:**
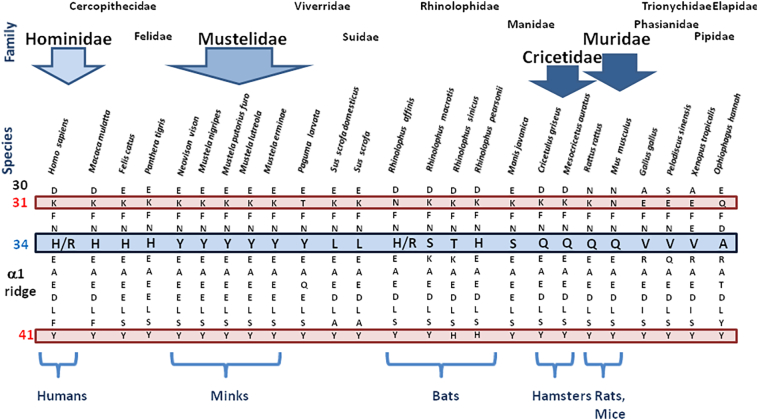
A comparison of ACE2 sequences from 24 different species. The species considered bling to belonging to the Hominidae, Cercopithecidae, Felidae, Lustelidae, Viverridae, Suidae, Rhinolophidae, Manidae, Muridae, Phasianidae, Trinychidae, Pipidae and Elapidae families. The multialignment was performed using Clustal Omega multiple sequence alignment. All sequences were obtained from the NCBI reference sequence database (see Materials and Methods) and were numbered according to amino acid position on the *Homo sapiens* ACE2 protein. In the schematic representation the comparison of the *Homo sapiens* ACE2 consensus protein and ACE2 orthologs was focused on sequence region 30–41, particularly the amino acid residue 34. The amino acids (single letter code) are in black. The amino acids at position 34 are highlighted in blue. Some of the amino acids known to be important for viral tropism are highlighted in red. The peptide sequence comparison includes *Homo sapiens* (human), *Macaca mulatta* (monkey), *Felis catus* (cat), *Panthera tigris* (tiger), *Neovison vison* (American mink), *Mustela nigripes* (black-footed ferret), *Mustela putorius furo* (ferret), *Mustela lutreola* (European mink), *Mustela erminae* (ermine), *Paguma larvata* (palm civet), *Sus scrofa* domestic (pig), *Sus scrofa* (boar), *Rhinolophus affinis* (bat), *Rhinolophus macrotis* (bat), *Rhinolophus sinicus* (bat), *Rhinolophus pearsonii* (bat), *Manis javanica* (pangolin), *Rattus rattus* (rat), *Mus musculus* (mouse), *Gallus gallus* (hen), *Pelodiscus sinensis* (turtle), *Xenopus tropicalis* (frog), and *Ophiophagus hannah* (snake). For complete sequences comparison of the ACE2 polymorphism among Chiroptera see [17]. For complete sequences comparison of the ACE2 polymorphism among Mustelidae see [31]. (For interpretation of the references to colour in this figure legend, the reader is referred to the web version of this article.)

Mutations in the spike gene and protein. No species-based segregation could be found between human and mink SARS-CoV-2 based on the presence of wild type or mutated Y453 or D614. Instead, viruses with identical residues 453 and 614 were found in both minks and humans indistinctly (Fig. 1). The distribution of the Y453F and D614G mutations in humans and minks has already been reported [17]. The Y453F mutation was found in both European minks (*M. lutreola*), American minks (*N. vison*) and humans. However, a clear geographic and species segregation was found with respect to associated mutations (Fig. 1). A series of mutations only associated with both Y453F and D614G was only found in humans in Denmark whereas they were absent in minks also bearing the double Y453F and D614G mutation in the same country (Table 1).

**Fig. 2:**
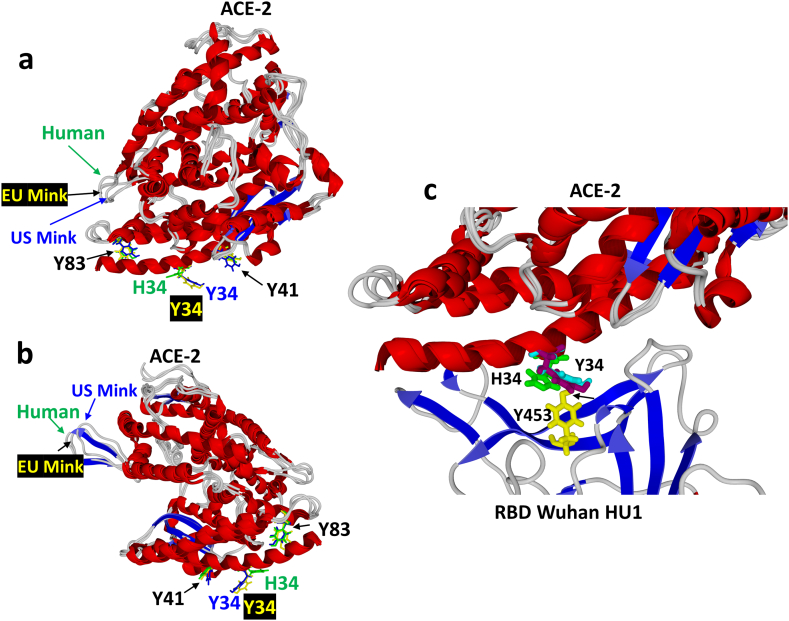
Structural homology between human and mink (EU and US) ACE-2. a-b. Superimposition of human and mink ACE2 structures. The models in panels a and b show two opposite views of superimposed structures of the N-terminal domain of human, European mink (Eu Mink)/*Mustela lutreola* and American mink (US Mink)/*Neovison vison* ACE2 with the location of H34 and Y34, as well as Y41 and Y83. H34 amino acid (human ACE2) is colored in green. Mink Y34 amino acid is colored in yellow (Eu Mink) and blue (US Mink). c. Representation of the conformation of RBD in interaction with the N-terminal domain of ACE2. In this representation ACE2 is above the SARS-CoV-2 RBD. H34 amino acid (human ACE2) is colored in green. Mink Y34 amino acid is colored in purple (Eu Mink) and cyan (US Mink). The potential clash between Y453 and Y34 (EU and US minks) is indicated by an arrow. A mutation of Y453 in the spike RBD is essential to avoid this clash. (For interpretation of the references to colour in this figure legend, the reader is referred to the web version of this article.)

Polymorphism in the ACE2 N-terminal region essential for binding to the viral spike. The ACE2 N-terminal region essential for the attachment of the viral spike from 23 species spanning from residue 30 to 41 was analyzed for polymorphism. The residues 31 and 41 have been reported as potentially playing a role in RBD binding specificity [31]. When comparing the RBD-binding region from humans and minks ACE2, a polymorphism could be seen at position 30 (D vs E), 34 (H vs Y) and 38 (D vs E). Aspartic acid (D) and glutamic acid (E) are two structurally very closely related amino acids bearing the same negative charge. However, the mutation at position 34 corresponded to a significant change with histidine (H) being present in the human ACE2 and tyrosine (Y), a larger amino acid, being found in the ACE2 protein from both European and American minks (Fig. 2a and b).

**Fig. 3:**
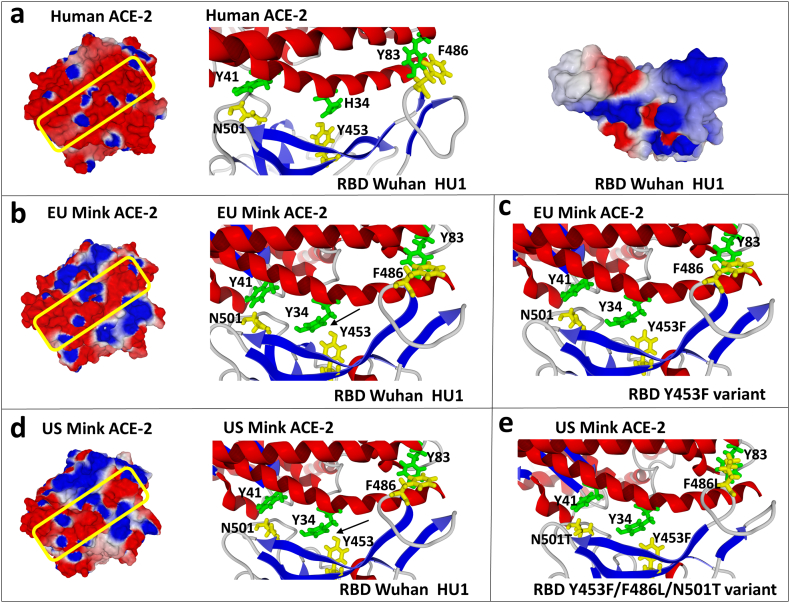
Interaction of human and mink ACE2 with the RDB in the viral spike of the Wuhan HU1 and selected variants of SARS-CoV-2. a. Human ACE2-RBD Wuhan HU1 complex. Left panel: The electrostatic surface potential of human ACE2 is mostly electronegative (red areas), with sparse electropositive spots (blue areas). The surface recognized by the RBD is indicated by a yellow frame. Middle panel: molecular complex between the Wuhan HU1 RBD and human ACE2. The N501 in the viral spike interacts with residue Y41 of ACE2. Y453 interacts with Y34, and F486 interacts with Y83. Right panel: electrostatic surface potential of the RBD. The electropositive areas are complementary to the electronegative interface of ACE2. b. EU mink AC2 interaction with the Wuhan HU1 RBD. Left panel: electrostatic surface potential of EU mink ACE2. It is clearly distinct from human ACE2 and more electropositive. Right panel: EU mink ACE2 interaction with the Wuhan HU1. c. EU mink ACE2 interaction with the Y453F variant. d. US mink AC2 interaction with the Wuhan HU1 RBD. Left panel: electrostatic surface potential of US mink ACE2. It is clearly distinct from both human ACE2 and EU mink ACE2. Right panel: EU mink ACE2 interaction with the Wuhan HU1. e. US mink ACE2 interaction with the Y453F/F486L/N501T variant. The H34 in human ACE2 is essential for the interaction with the Y453 of the SARS-CoV-2 Wuhan HU-1 reference strain (a). The molecular models (b-e) Y34 in minks ACE2 causes a conflict when approaching the viral spike RBD. Specifically, the OH group of Y453 is too close from Y34 (arrows in b and d), which is obviously not the case when Y453 faces H34 in the human ACE2 (a). The Y453F found in mink SARS-CoV-2 eliminates this conflict (c and e). (For interpretation of the references to colour in this figure legend, the reader is referred to the web version of this article.)

Structural changes associated with interspecies transmission. The passage of SARS-CoV-2 from humans to minks was accompanied by a mutation from a tyrosine (Y) to a phenylalanine (F) in the RBD at position 453 [43]. When bound to ACE2, the spike residue 453 is facing the residue 34 in the ACE2 RBD-binding domain (Fig. 2c). The residue 453 in the RBD of human strains of SARS-CoV-2 was a tyrosine which, when facing the histidine present at position 34 in the human ACE2, generated an optimal interaction. Indeed, the oxygen atom borne by the phenolic group of tyrosine was at 2.1 Å from one of the protonated atoms of nitrogen of the imidazolium group, consistent with the establishment of a H-bond. The mobility of the histidine ring was facilitated by the CH_2_ group of H34, allowing an appropriate orientation of H34 and Y453 side chains (Fig. 3a). The superimposition of structures of the N-terminal domain of human ACE2 with ACE2 from *M. lutreola* and *N. vison,* indicated that the folding of the ACE2 orthologs was globally similar to that of the human ACE2, despite many substitutions (Fig. 2a and b). However, the electrostatic surface potential of EU mink ACE2 and US mink ACE2 slightly differed from that of the human ACE2 which was more electronegative (Fig. 3a, b and d). Incidentally, when Y453 was facing another tyrosine at position 34 in the mink ACE2, a steric conflict occurred preventing the establishment of the stabilizing interaction between the RBD and its receptor (Fig. 2c). Indeed, the oxygen atom of the phenolic group of Y453 was literally touching the aromatic ring of Y34 in mink ACE-2. The mutation Y453F observed in mink strains of SARS-CoV-2 suppressed the clash and restored the optimal binding with the mink ACE2 Y34 residue (Fig. 3b, d and e). In this case, both aromatic rings adopted a perpendicular orientation characteristic of T-shaped CH-pi stacking. Distance measurements revealed that the aromatic side chains of Y34 and F453 were separated by 3 Å, a distance fully consistent with this type of interaction.

**Fig. 4:**
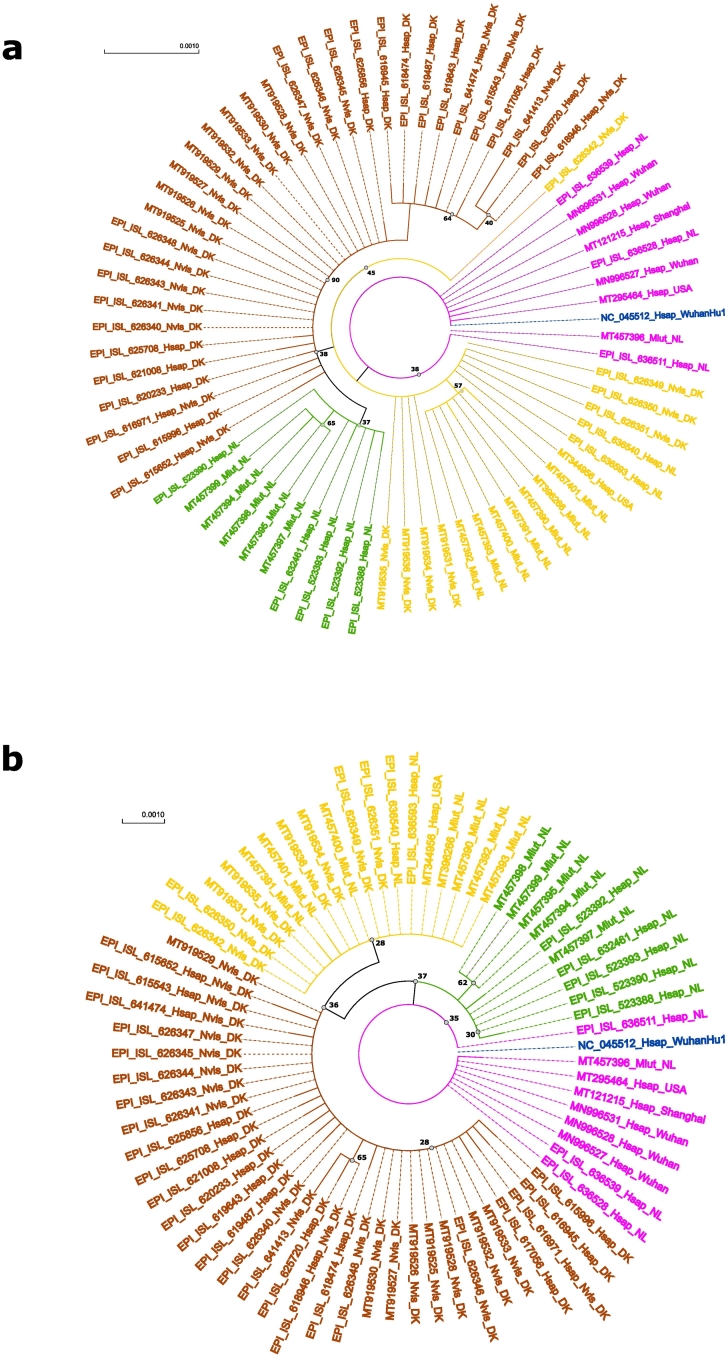
Maximum likelihood phylogenetic trees of the SARS-CoV-2 spike gene and protein from different isolates. Sample names are built with the Genbank of GISAID accession number followed by a four-letter code (Mlut for *Mustella lutreola*, Nvis for *Neovison vison* and Hsap for *Homo sapiens*) identifying the species and a country code (DK for Denmark, NL for The Netherlands and USA for United States of America) indicating the geographical origin of the sample except for the sequences from China in which the city of origin is indicated. The tree was rooted using the spike gene sequence of the Wuhan Hu1 SARS-CoV-2 strain. The colour code corresponds to the nature of the 453 and 654 residues. Blue: Outgroup rooting the tree. Purple: Y453 + D614. Yellow: Y453 + D614G. Green: Y453F + D614. Brown: Y453F + D614G. a) Maximum likelihood (HKY) phylogenetic tree of the SARS-CoV-2 spike genes. b) Maximum likelihood (Q.plant + G + F) phylogenetic tree of SARS-CoV-2 spike. (For interpretation of the references to colour in this figure legend, the reader is referred to the web version of this article.)

**Fig. 5:**
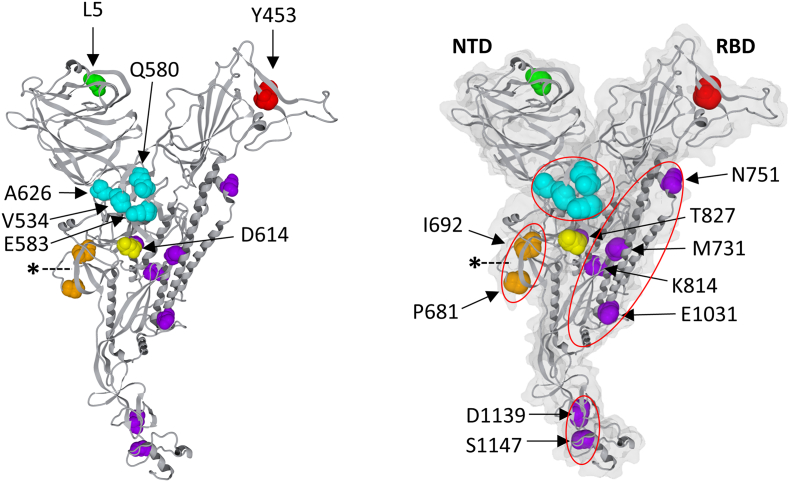
Position of the principal mutations in the SARS-CoV-2 spike protein. For clarity, two views of the spike protein are shown, one in ribbons (left panel), the other one in ribbon + surface rendition (right panel). A colour code indicates the localization of the mutations: green, signal sequence; red, RBD; orange, cleavage site (*); cyan, amino acid residues involved in the conformational change that demasks the RBD; purple, amino acid residues involved in the fusion machinery. (For interpretation of the references to colour in this figure legend, the reader is referred to the web version of this article.)

Compensatory mutations in the spike protein of SARS-CoV-2 strains. 16 strains of SARS-CoV-2 bearing both the mink-related Y453F mutation and one or more additional Spike mutations have been already described [17]. A total of 18 mutations additional to Y453F and D614G were observed. Interestingly these mutations were found only in Denmark following bidirectional infections between humans and American minks (*N. vison*) in rearing (Table 1, Fig. 4). Five mutations, i.e. N751Y, I692V, D1139Y, S1147L and M1229Y were found on viruses isolated both from humans and minks. The mutations I692V and M1229Y were found in the same strain (EPI_ISL_615652) whereas the other three mutations were individual, each one being present in a different strain (EPI_ISL_641474, EPI_ISL_618946 and EPI_ISL_616971). Only one case of additional mutations was found in a SARS-CoV-2 strain isolated from minks only. It is the triple L5F-N751Y-C1250F in strain EPI_ISL_641413. The N571F mutation was found both in humans and minks whereas all the other 11 mutations were found only in SARS-CoV-2 strains isolated exclusively from humans. With the exception of the double A626S-N751Y mutation in the strain EPI_ISL_625720, all mutations were singletons in different human-isolated SARS-CoV-2 strains. The 3-D positioning of these mutations indicated that they were distributed in five different functional groups (Fig. 5): i) the signal sequence, which is no longer present in the NTD, ii) the RBD, which interacts with ACE2; iii) amino acids involved in the conformational change that demasks the RBD, iv) amino acids involved in the S1-S2 cleavage site and v) amino acids involved in membrane fusion. This indicated that these mutations were not random. Except for Y453 in the RBD, none of these mutations were directly involved in ACE2 interaction and binding suggesting that they might be involved in a change of kinetic of the interaction, i.e. facilitate the occurrence of the binding, rather than in affinity per se. The mutation L5F is located in the signal peptide and thus is no longer present in the mature protein.

Deletions in the spike N-terminal domain (NTD). In addition to single point mutations, the NTD of mink spike proteins is also characterized by the deletion of H69 and V70. This deletion, which is commonly found in the B.1.1.7 variant, compacts the NTD and renders its surface more electropositive [36]. This double deletion optimizes the initial interaction of the spike protein with lipid rafts, which display an electronegative surface potential.

## 4 Discussion

Although it cannot be absolutely ruled out that the outbreak of SARS-CoV-2 in certain mink farms could have been initiated with the introduction into the farm of a sick or asymptomatic carrier animal (e.g., wild Mustelidae which sometimes enter farms to steal food) [44,45], whole genome sequencing of viruses and phylogenic analysis evidenced that minks in Northern Europe were infected by staff members in charge of animal care and/or cage maintenance. Indeed, mink SARS-CoV-2 genomes available through the GISAID database indicated that they branched on human SARS-CoV-2 sequences and that mutants including the Y453F change occurred afterwards [14]. The virus spreading and the accumulation of host-specific mutations were favored in mink farms by intensive breeding conditions with overcrowded animals caged maintained in poorly ventilated rooms.

This work demonstrates the pivotal role of the ACE2 amino acid 34 and the S protein amino acid 453 in the spike-ACE2 interaction during SARS-CoV-2 infection. Two spike protein mutations have previously been described as specific from SARS-CoV-2 circulating in minks, i.e. S1147L and Y453F [17]. The S1147L mutation is located very far from the site of interaction with ACE2. Y453F is the only mutation located in the RBD region, thus in direct interaction with ACE2. This mink-associated mutation of SARS-CoV-2 in which the tyrosine (Y) present in the human viral strains is replaced by a phenylalanine (F) plays a key role in the affinity of the RBD for the mink ACE2. The ACE2 amino acid 34 in minks is characterized by the presence of a tyrosine (Y) whereas it is a histidine (H) in humans. The H34Y substitution with respect to human ACE2 is present in all species of Mustelidae for which ACE2 ortholog sequence was available [17]. This amino acid difference is crucial since the tyrosine at position 34 in the mink ACE2 results in a conflicting interaction of polar groups and a reduced affinity when facing the tyrosine present at position 453 in human SARS-CoV-2 strains. However, the replacement of the tyrosine by a phenylalanine at position 453 the S protein from mink-isolated SARS-CoV-2 strains is eliminating this conflict and is restoring the ACE2-RBD affinity through an ACE2 tyrosine-spike phenylalanine interaction. This explains the selection of this mutation in minks SARS-CoV-2 strains. However, bidirectional human and mink cross-species SARS-CoV-2 infections have been demonstrated [14]. Therefore, viral strains bearing the Y453F mutations originating minks have been infecting humans back [17]. In this situation, the conflict between ACE2 amino acid 34 and SARS-CoV-2 spike amino acid 453 is present again since this time H34 is facing F453. In humans, a linear peptide comprising the Y453 amino acid was reported as acting as a T-cell presenting epitope HLA-DPA1 [46]. The amino acids L452 and R454 flanking Y453 are involved in discontinuous B-cell epitopes [47]. A VSV-SARS-CoV-2 wild type spike put in presence of the RBD-binding REGN10933 monoclonal antibodies in vitro led to the selection of the Y453F mutation conferring resistance to this antibody [48]. This could also occur in minks. Interestingly, a series of additional mutations in the SARS-CoV-2 spike have been observed in infected humans along with the mutation Y453F. These mutations did not intervene in the ACE2-RBD binding process but were located in regions involved in the modification of structure-function properties of the S protein. The fact that all these mutations are gathered in specific protein domains with a role in the kinetic of binding reaction is not a coincidence but rather the result of a selective pressure. The S1-S2 cleavage spike region is playing a key role in potentiating the membrane fusion process and therefore increasing the overall kinetics of interaction [49]. The position of these mutations follows a functional logic that translates the global fusion mechanism of SARS-CoV-2 into key amino acid changes. The deletion in the NTD has a major impact on the kinetics of virus binding to the plasma membrane of host cells as it induces a compaction of the domain and a concomitant increase of the electrostatic surface potential. As the NTD interacts with the electronegative surface of lipid rafts, the consequence of this deletion is an improved access to these ganglioside-rich domains of the plasma membrane. Hence, those viruses with an increased electrostatic potential in the NTD have a kinetic advantage over competitors [36]. This evolution of SARS-CoV-2 has led to a progressive increase of the surface potential that has culminated with Delta variants. The other mutations are logically found in the SARS-CoV-2 Spike RBD (Y453) as the result of virus adaptation to mink hosts, but also in domains of the spike protein involved in the conformational change inducing the demasking of the RBD (e.g. amino acid 583 which is close to the quasi-universal mutation D614G), in the proteolytic cleavage site (amino acids 681 and 692, but also V308 which is close to this site in the 3D structure of the spike protein) and in the rod-like region, i.e. alpha-helical rod-like regions involved in the fusion process (e.g. amino acids 731 and 1031).

**Supplementary Fig. 1:**
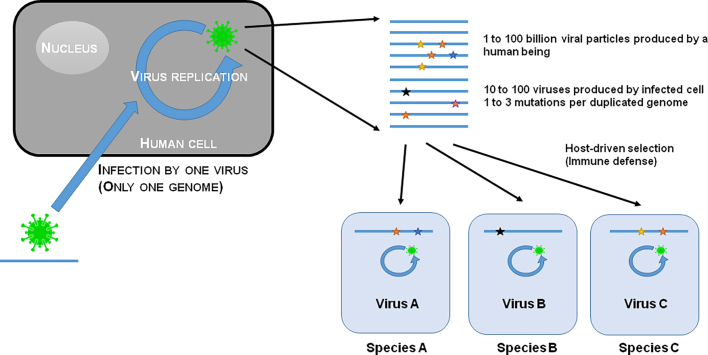
Schematic representation of the quasispecies evolutionary process.

These results shed light on an important phenomenon in RNA virus evolution and emergence of infectious diseases, i.e. the host driven evolution and quasispecies adaptation of these viruses. Like all RNA viruses, SARS-CoV-2 is evolving through the quasi-species mechanism [[50], [51], [52]] process (Supplementary Fig. 1. The quasispecies mechanism of evolution is based on the production at each generation of a very high number of virions, each one differentiating from the others by a very small number of mutations. Most of these mutants are not viable. However, some, by chance, carry mutations allowing them to be more transmissible or to overcome the host defenses and thus to survive. These mutants will generate the next generations of virions. Overtime, owing to the selective pressure imposed by the host itself, or host-driven selective pressure, very adapted virus lineages will be selected. This mechanism of evolution allows the virus to permanently adapt to its host, to the evolution of the host defenses and even to vaccines. This is clearly exemplified by the succession of SARS-CoV-2 variants observed since the first description of this virus. There is thus no preadaptation and no preexisting mutation favoring specificity to a given host. Variant viruses emerge post-infection under positive selective pressure (i.e. host-driven selective pressure) specific to mink host defense mechanisms [[53], [54], [55]] as well as restriction factor and/or viral receptor/co-receptor adaptation. The mutations observed in the virus genome reflect this adaptation to the host. A virus will evolve differently and acquire different mutations in different hosts in order to specifically adapt to these hosts [7,56]. There is no definitive mutation but rather a dynamic and permanent process of adaptation and thus moving from one host to another, SARS-CoV-2 will quickly acquire or lose specific mutations. This dynamic process of adaptation is exemplified in the human-mink interspecies infection context. The human SARS-CoV-2 acquired in mink a mutation on the amino acid 453 to solve the conflicting interaction with the ACE2 Y34. During the reverse infection of humans by mink-adapted-SARS-CoV-2 a reverse adaptation occurred but not through a reverse mutation from the spike protein amino acid 453 to tyrosine but by the accumulation compensatory mutations in other domains of the protein having a potentiating effect on the dynamic of the virus-cell interaction. The lack of reverse mutation might be explained by a lesser conflict between H34 and F453 than between Y34 and Y453. Nevertheless, the Y453F mutation did not remain in the human population after minks were isolated and slaughtered [17] indicating that a reverse mutation has occurred which was more stable on humans or that mink-mutated variants were outcompeted by human variants and disappeared.

A similar scenario was described after the discovery of SARS-CoV-2-infected hamsters in Hong Kong [15,57]. A hamster-derived SARS-CoV-2 Delta variant was able to infect humans back and to undergo human to human transmission [15]. Specific mutations were described in this hamster-adapted Delta variant, three of them being located on the Spike protein. Two of the mutations, L18F and H49Y, were located in the N-terminal domain. The mutation D427G was located inside the RBD but outside the Receptor Binding Motive (RBM) which interacts directly with the ACE2 receptor. Another hamster-specific mutation, T38I, was located in ORF10. The D427G mutation is of particular importance in both hamsters and humans [57]. In the hamster ACE2 protein, H34 is replaced by Q34 which can still interact with Y453 through a H-bond. However, this generates a torsion pushing the amide group of Q34 to a direction opposite to that with H34. The D427G mutation annihilates this structural conflict. This mutation breaks the H-bond between D427 and G413. The α-helix is degraded into a more flexible loop leading to a NH-π interaction between the residue N422 to and the aromatic ring of Y453. Y453 is therefore attracted by the RBD, allowing the space required for the side chain of Q34 to recover the initial orientation found with H34. This stabilizes the H-bond between Y453 and Q34 and reduce the distance from Q34 to 2.7 Å to 1.6 Å for D427 and G427, respectively. This conformational advantage is still present in humans in whom he H-bond between Y453 and H34 remains optimized. The distance to Y34 moves from 3.5 Å to 2.7 Å and 2.2 Å for D427 and G427, respectively [57]. The conformational change is also impacting the aromatic ring of the RBD F486, restoring the optimal energy of interaction [57]. A perhaps similar situation might exist with Omicron (BA.1). The spike protein of this lineage is believed to have been subjected to a strong positive selection from a different host species [58]. The mutations in the omicron RBD correspond from many of them to those needed for adaptation to mice, in particular those involved in the affinity of the spike RBD for the mouse ACE-2. An ancestral human lineage of Omicron may very well have infected mice before evolving specifically in this rodent species from and infecting back humans as the Omicron lineage [39,58]. The mutational pattern of the omicron spike protein is more likely to be the result of a series of back and forth human/mouse contaminations rather than a gradual evolution of SARS-CoV-2 in human hosts [39].

The infection of pets, like cats and dogs, from human owners has been reported [[8], [9], [10]]. However, there is no report of reverse infection from these animals to humans. One hypothesis can be that cat- or dog-acquired mutations are less efficient in humans, making the human back infection less likely. There is however another parameter to consider which is the population density. The species from which SARS-CoV-2 infected humans back, i.e. minks and hamsters, are found in mass rearing where the virus population can be amplified giving rise to numerous mutations and high genetic diversity. Furthermore, these rearing are commercial activities and other people than caretakers, like customers or sellers can be infected. This is what happened with hamsters in Hong Kong [15]. The situation with pets is highly different. There is no mass density. There is usually one or few owners, a family, with one or few pets. There is thus no ground for amplification of the viral population, which is also a key element in the emergence of clusters within the human population itself. Besides, the initial contamination of pets comes from the owner, who is by definition already contaminated by the same virus making it extremely difficult to trace any back contamination. Contaminations to other species occur probably a lot more than suspected but it is essentially with animals present the immediate human vicinity.

The occurrence of compensatory mutations and mechanisms is also giving an insight on the process of emergence of infectious diseases like COVID-19. Optimal affinity and adaptation are not required for infection. A virus can infect a host for which only the minimal affinity necessary to ensure interaction. An optimal affinity is not necessary at first and will nevertheless be acquired by adaptive mutations through quasispecies evolution. A virus can thus remain in a host for a long time at a “suboptimal stage” before reaching the level of affinity and transmissibility required to trigger an epidemic. This also allows RNA viruses to easily move from one host to another without any mechanism such as “species barrier crossing”. It also allows viruses to permanently adapt to the host defense build-up through successive selection of variants, a process well exemplified in COVID-19. The pivotal role of ACE2 amino acid at position 34 and Spike amino acid at position 453 and the ability to develop compensatory mutations to ensure optimal affinity are essential to the pathogenicity of SARS-CoV-2 but also to the initial process of emergence and to its sustainability through variants. This key role will be essential to investigate in order to better understand life history of the virus and to develop proper prophylactic approaches.

The following are the supplementary data related to this article.

## Author contributions

All authors contributed towards conceiving the manuscript. NY and JF performed the structural analysis; RF performed the virus genome phylogenetic analysis; CD performed the multiple sequence alignment. RF and CD wrote the first draft of manuscript. All authors reviewed and approved the final version of the manuscript.

## Funding

This work was supported by the French Government under the « Investissements d'avenir » (Investments for the Future) programme managed by the 10.13039/501100001665Agence Nationale de la Recherche (ANR, FR: National Agency for Research), (reference: Méditerranée Infection 10-IAHU-03) to Prof. Didier Raoult and annual budget allocation from Aix-Marseille Université and IRD to the MEPHI laboratory.

## Ethical approval

None required.

## Declaration of Competing Interest

The authors declare that the research was conducted in the absence of any commercial of financial relationships that could be construed as a potential conflict of interest.
